# Spina Bifida Occulta with Bilateral Spondylolysis at the Thoracolumbar Junction Presenting Cauda Equina Syndrome

**DOI:** 10.1155/2020/2425637

**Published:** 2020-01-13

**Authors:** Kentaro Mataki, Masao Koda, Yosuke Shibao, Hiroshi Kumagai, Katsuya Nagashima, Kousei Miura, Hiroshi Noguchi, Toru Funayama, Tetsuya Abe, Masashi Yamazaki

**Affiliations:** Department of Orthopedic Surgery, Faculty of Medicine, University of Tsukuba, 1-1-1 Tennodai, Tsukuba, Ibaraki 305-8575, Japan

## Abstract

Several reports have described the coexistence of spina bifida occulta (SBO) and spondylolysis, but the majority of defects occur at L5. No report has described the coexistence of SBO and spondylolysis at the thoracolumbar junction. We report a case of SBO with spondylolysis at L1, presenting cauda equine syndrome. A 37-year-old man presented with a gait disorder as a result of bilateral motor weakness of the lower extremities. A plain radiograph showed local kyphosis at L1-2 as a result of severe degenerative change and wedging of the vertebral body at L1. Magnetic resonance imaging (MRI) revealed degenerative disc changes and severe canal stenosis at L1-2. Computed tomography (CT) revealed SBO and spondylolysis at L1. He was diagnosed with cauda equina syndrome related to SBO and spondylolysis at L1. Posterior interbody fusion and decompression at L1-2 were performed. After surgery, his muscle power recovered to normal strength. The possible mechanisms in this case are the strain on anterior elements as a result of disruption of the posterior elements due to SBO and spondylolysis. The coexistence of SBO and spondylolysis at the thoracolumbar junction might induce at-risk status of increased strain to the anterior elements that may cause cauda equina syndrome.

## 1. Introduction

Spina bifida occulta (SBO) is a common malformation of the lamina of the spine, most commonly occurring in the sacrum or lower lumbar spine [1, 2]. Spondylolysis is a common etiology of back pain in children and adolescents. SBO is associated with spondylolysis of the lumbar spine in 11.8-35% of patients [3, 4]. There are several reports of the coexistence of SBO and spondylolysis; the majority occur at L5 [2, 5]. However, no report has described the coexistence of SBO and spondylolysis at the thoracolumbar junction.

The aim of this paper is to describe a clinical example of treatment for cauda equina syndrome as a result of the coexistence of SBO and spondylolysis at the thoracolumbar junction.

## 2. Case Report

A 37-year-old man presented with progressive limitation of activities as a result of bilateral motor weakness of the lower extremities for several weeks. He had no history of strenuous sporting activity or low back pain. Neurological examination revealed motor weakness in iliopsoas and quadriceps muscles, and muscle power was rated at a manual muscle testing level of 2. He had no urinary dysfunction or lower extremity sensory dysfunction.

**Figure 1:**
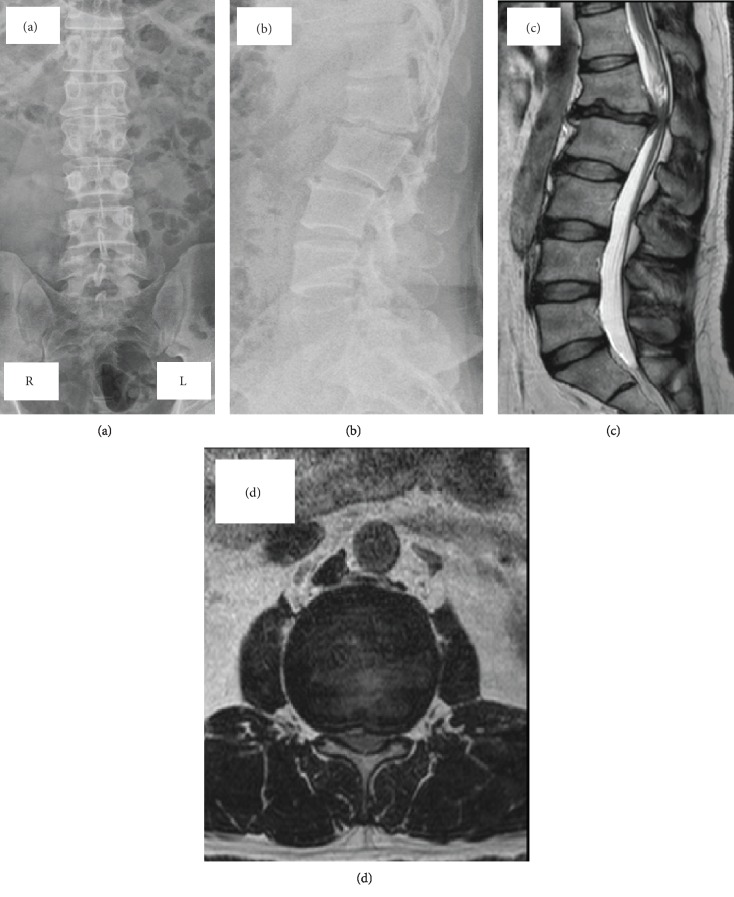
Anterior-posterior radiograph (a) and lateral radiographs (b) of the lumbar spine showing severe degenerative change at L1-2 and compression of the L1 vertebral body. Sagittal (c) and axial (d) T2-weighted magnetic resonance images showing degeneration of the disc and severe canal stenosis at the L1-2 level. R indicates right side; L, left side.

**Figure 2:**
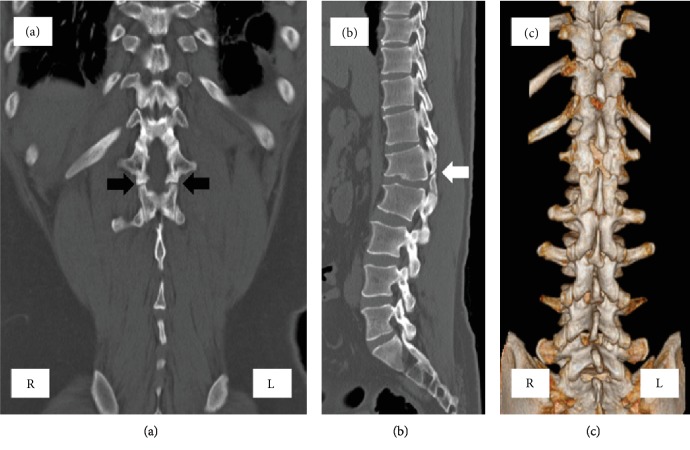
Computed tomographic (CT) scan. Coronal view (a) revealed bilateral spondylolysis (black arrow) and a bony defect of the lamina at L1. A sagittal view (b) revealed spondylolysis (white arrow) at L1. Three-dimensional reconstruction of the computed tomography scan (c) showing the coexistence of spina bifida and spondylolysis at L1. R indicates right side; L, left side.

A plain radiograph showed local kyphosis at L1-L2 as a result of severe degenerative change at the L1-2 disc level and wedging of the vertebral body at L1 (Figures 1(a) and 1(b)). Magnetic resonance imaging (MRI) revealed severe degenerative disc change and severe canal stenosis at L1-2 (Figures 1(c) and 1(d)). Computed tomography (CT) revealed SBO and spondylolysis at L1 and no other malformations (Figures 2(a)–2(c)).

**Figure 3:**
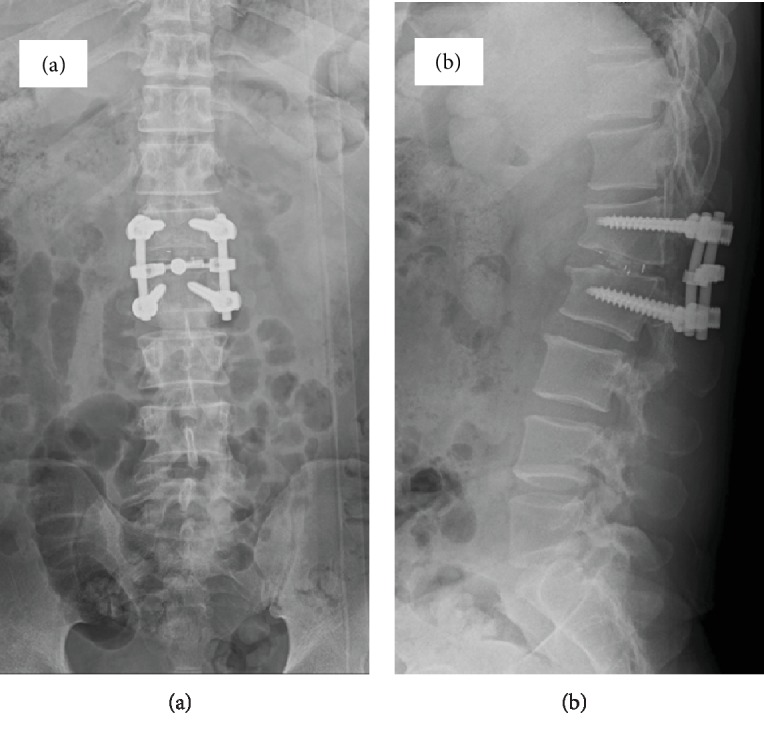
Postsurgery radiographs. Anteroposterior (a) and lateral (b) views, showing instrumented posterolateral interbody fusion at L1-2.

He was diagnosed with cauda equina syndrome at the L1-2 level related to SBO and spondylolysis at L1. Posterior lumbar interbody fusion at L1-2 was performed (Figure 3). After surgery, his symptoms improved promptly, and he was able to walk with a cane on his discharge.

## 3. Discussion

SBO is caused by failure of fusion between posterior vertebral elements without affecting the spinal cord or meninges. It is usually observed at L5 and/or at the upper one or lower two sacral vertebrae [1, 2]. Goto et al. reported a case of SBO at the thoracolumbar junction and estimated the incidence as <5% within all SBO patients [6, 7]. SBO is associated with spondylolysis and spondylolisthesis of the lumbar spine [4, 8]. SBO occurs with spondylolysis of the lumbar spine in 11.8-35% of patients [3], and in one-third of patients with the isthmic type of spondylolisthesis [8].

The presence of dysplastic or disrupted posterior elements in SBO may increase load on the pars interarticularis. Sakai et al. reported that the incidence of spondylolysis was significantly higher in patients with SBO than in those without SBO (odds ratio = 3.7) [4].

The coexistence of SBO and spondylolysis occurs at L5 in the majority of cases [2, 5]. To our knowledge, no report has described the coexistence of SBO and spondylolysis at the thoracolumbar junction.

We hypothesize that the possible mechanism for this is that the disruption of the posterior elements caused by SBO and spondylolysis increases strain on the anterior elements, such as the L1-2 disc and L1 vertebral body.

In our case, the etiology of the spondylolysis remains unclear because lumbar spondylolysis had not been diagnosed until the patient's first visit to our hospital. The patient had no history of low back pain as an adolescent. This patient required immediate decompression and fusion surgery because of neurological deficits due to cauda equina syndrome. Several cases of cauda equina syndrome caused by L1-2 disc hernia have been reported [9, 10]. In this case, because of the disruption of the posterior elements caused by SBO and spondylolysis, we performed posterior lumbar interbody fusion at L1-2.

Kumar et al. reported that there is a significant risk of nerve root damage during surgical exposure due to defects of the posterior elements in patients with such a coexistent lesion. Therefore, it is mandatory to be examined prior to surgery by pelvic outlet views using CT [5]. With preoperative images including MRI and CT, we were aware of the coexistence of SBO and spondylolysis at the thoracolumbar junction before surgery in the present case.

## 4. Conclusion

The coexistence of SBO and spondylolysis at the thoracolumbar junction might induce at-risk status of increased strain to the anterior elements of the spine that may cause cauda equina syndrome.

## Additional Points

*Highlights*. Spina bifida occulta (SBO) is associated with spondylolysis of the lumbar spine. The coexistence of SBO and spondylolysis often occurs at L5. No report has described those disruptions at L1 presenting cauda equina syndrome. Defects of the posterior elements might increase the strain on the anterior aspect. The coexistence of SBO and spondylolysis may cause cauda equina syndrome.
